# All-cause mortality after major gastrointestinal bleeding among patients receiving direct oral anticoagulants: a protocol for a systematic review and meta-analysis

**DOI:** 10.1186/s13643-022-02146-5

**Published:** 2022-12-13

**Authors:** Nicholas L. J. Chornenki, Tobias Tritschler, Fabian Stucki, Roupen Odabashian, Jenneke Leentjens, Faizan Khan, Valentina Ly, Deborah M. Siegal

**Affiliations:** 1 Internal Medicine Residency Program, Queens University, Kingston, ON Canada; 2 Department of General Internal Medicine, Inselspital, Bern University Hospital, University of Bern, Bern, Switzerland; 3 Department of Medicine, University of Ottawa, Ottawa, ON Canada; 4 Clinical Epidemiology Program, The Ottawa Hospital Research Institute, Ottawa, ON Canada; 5 Radboud University Medical Center, Radboud University, Nijmegen, Netherlands; 6 School of Epidemiology and Public Health, University of Ottawa, Ottawa, ON Canada; 7 Health Sciences Library, University of Ottawa, Ottawa, Canada

**Keywords:** Bleeding, Direct oral anticoagulants, Mortality, Anticoagulation

## Abstract

**Background:**

Gastrointestinal (GI) bleeding represents the single most frequent site of anticoagulant-related bleeding. Adverse outcomes after major GI bleeding including mortality are not well characterized and, as a result, may be underappreciated in clinical practice. We aim to conduct a systematic review and meta-analysis of the risk for 30-day all-cause mortality after major GI bleeding among patients receiving DOACs.

**Methods:**

Electronic databases including MEDLINE, EMBASE, and Cochrane CENTRAL will be systematically searched to identify randomized controlled trials and prospective and retrospective cohort studies reporting 30-day all-cause mortality in adults with DOAC-related major GI bleeding. At least two investigators will independently perform study selection, risk of bias assessment, and data extraction. The proportion of deaths following a major GI event relative to the number of major GI bleeding events will be calculated for each individual study, and results across studies will be pooled using random-effects meta-analysis. We will assess risk of bias using criteria proposed by the GRADE group for prognostic studies.

**Discussion:**

The findings of this systematic review and meta-analysis will provide clinicians and patients with estimates of mortality after the most common major bleeding event to support shared decision making about anticoagulation management.

**Trial registration:**

PROSPERO CRD42022295815.

## Background

Like all anticoagulants, the use of direct oral anticoagulants (DOACs; apixaban, rivaroxaban, edoxaban, dabigatran) for prevention and treatment of thromboembolism is limited by bleeding. About 2 to 4% of anticoagulated patients experience major bleeding each year and another 10 to 12% experience clinically relevant non-major bleeding [1–8]. Unlike intracranial bleeding which is a rare severe event associated with substantial mortality and long-term morbidity, gastrointestinal (GI) bleeding is the most frequent single site of anticoagulant-related bleeding representing up to 40% of bleeds [9–11].

As a class, DOACs increase rates of GI bleeds, the adverse outcomes of which, including mortality, have been less well characterized compared to intracranial bleeds [12, 13]. This is due, at least in part, by a lack of standardized and validated definitions for fatal GI bleeding which may not reflect the potential severity of events. Overall mortality incorporates bleeding and non-bleeding causes of death and appears to be as high as 10% after major GI bleeds [14–17]. In a sub-study of the ARISTOTLE (Apixaban for Reduction in Stroke and Other Thromboembolic Events in Atrial Fibrillation) trial, all-cause mortality was 9.2% within 30 days of major extracranial bleeding, the majority of which was GI bleeding [14]. Further, patients with major extracranial bleeding had a 12-fold increased risk of all-cause death in comparison with those without such bleeding [14]. Similar rates of 30-day all-cause mortality (10%) were reported among DOAC-treated patients with acute major GI bleeding treated with specific DOAC reversal agents [15–17].

By summarizing the available evidence regarding mortality after DOAC-related GI bleeding, this systematic review and meta-analysis will provide key prognostic information for shared decision-making between patients and their providers, and provide researchers with prognostic estimates to support prospective prevention and treatment studies.

## Objective

The primary objective is to determine the risk of all-cause mortality within 30 days of major DOAC-related GI bleeding.

## Methods

This protocol has been developed following the Preferred Reporting Items for Systematic Review and Meta-Analysis Protocols (PRISMA-P) statement [18] and prospectively registered with PROSPERO (CRD42022295815). Important amendments made to the protocol will be documented and published alongside the results of the final systematic review. Reporting of the final systematic review will follow the PRISMA statement [19].

### Search strategy and study selection

The search strategy was developed and peer-reviewed by at the University of Ottawa [20] and includes MEDLINE (Ovid), Embase (Ovid), Cochrane Central Register of Controlled Trials (Ovid), CINAHL (EBSCO), and Web of Science (Core Collection) (see Appendix 1 for full search details) with no limits to language or publication date. The main search concepts included terms related to atrial fibrillation or venous thromboembolism, GI hemorrhage, and DOACs. Search results will be exported to Covidence (Melbourne, Australia) and duplicates will be removed. Title and abstract screening and full-text review of potentially eligible studies will be conducted independently by two reviewers. Disagreements will be resolved by discussion, or involvement of a third reviewer if consensus cannot be reached among the two reviewers.

### Study inclusion criteria

Studies will be eligible for inclusion if they meet the following criteria.

#### Population

Adults (18 years or older) treated with an oral anticoagulant for atrial fibrillation or venous thromboembolism who experienced a major GI bleeding event. Venous thromboembolism will be defined as lower limb deep vein thrombosis (DVT), pulmonary embolism (PE), or both. Studies which included individuals treated for non-limb venous thrombosis (e.g., cerebral vein, splanchnic vein) in addition to DVT and/or PE will be eligible. Studies which included only patients with non-limb venous thrombosis will not be eligible. For the purpose of this review, major bleeding will be defined as per individual studies. Hospitalization for GI bleeding will also be considered major GI bleeding.

#### Intervention and comparator

The review will include studies wherein participants received anticoagulation with an approved DOAC (i.e., rivaroxaban, apixaban, dabigatran, and edoxaban). Studies will be excluded if they exclusively evaluate anticoagulants other than DOACs. Studies including participants treated with antiplatelet agents in addition to anticoagulants (double or triple therapy) will be eligible for inclusion. Since the aim of the review is to determine the prognosis (mortality) of patients with a major GI bleeding event on DOACs, a comparator is not applicable.

#### Outcome

Studies that report all-cause mortality after a major GI bleed will be eligible.

#### Study design

Randomized controlled trial (RCT), and observational (prospective and retrospective) studies will be eligible. Case reports, cross-sectional studies, and case series will be excluded.

### Primary outcome

The primary outcome is 30-day all-cause mortality after major GI bleeding; measured by the number of deaths within 30 days following a major GI bleeding event relative to the number of major GI bleeding events.

### Secondary outcomes

Secondary outcomes include the number of deaths (a) during the entire follow-up and (b) within 90 days following a major GI bleeding event, and number of fatal GI bleeding events (as defined in individual studies) relative to the number of major GI bleeding events.

### Data extraction

Data will be extracted independently in duplicate using a standardized data extraction form, with discrepancies resolved by the same approach as for disagreements in the study selection process. Data extracted will include the information in Appendix 1. For studies which report bleeding outcomes within a combined cohort of DOAC and VKA-treated patients, we will use only DOAC treated patients.

### Handling of duplicate and missing data

For studies which reported findings from the same study participants in more than one publication, data will be extracted from the most complete data set, including information from other publications where needed. If studies include both VKA- and DOAC-treated patients and DOAC-specific data cannot be extracted, study authors will be contacted. Studies for which DOAC-specific data cannot be provided will be ineligible. We will not contact study authors for other missing or incompletely reported data.

### Risk of bias assessment

Risk of bias assessment will be conducted by two reviewers independently. As our analysis is concerned with prognosis (all-cause mortality after major GI bleeding) the risk of bias of included studies will be assessed using modified criteria proposed by Grades of Recommendation, Assessment, Development, and Evaluation (GRADE) for assessing the risk of bias in prognostic studies (Tables 1 and 2) [21]. Individual arms of RCTs will be treated as prospective cohorts for the analysis. Studies with one or more domains marked as “definitely no” or two or more domains marked as “definitely no” or “probably no” will be judged as being at high risk of bias. In order to be considered at low risk of bias all domains would need to be judged as “probably yes” or “definitely yes”. Otherwise, the study will be considered as having an unclear risk of bias. Disagreements will be resolved by discussion and an attempt at consensus. Involvement of a third reviewer will be used if consensus cannot be reached among the original two reviewers.

**Table 1:** Summary of the bias domains and prompting items for risk of bias assessment

	**Bias domains**		
	**1. Study participation**	**2. Study attrition**	**3. Outcome measurement**
Optimal study or characteristics of unbiased study	The study sample adequately represents the population of interest	The study data available (i.e., participants not lost to follow-up) adequately represent the study sample	The outcome of interest is measured in a similar way for all participants
Criteria for assessment of risk of bias	Was there a representative and well-defined sample of patients? - Who did not have the outcome of interest at the time of initial observation? - Who were at a similar, identifiable, common, and possible early point in the course of the disease?	Was follow-up sufficiently complete?	Were objective and unbiased outcome criteria used?
	• Definitely yes (low risk of bias) • Probably yes • Probably no • Definitely no (high risk of bias)	• Definitely yes (low risk of bias) • Probably yes • Probably no • Definitely no (high risk of bias)	• Definitely yes (low risk of bias) • Probably yes • Probably no • Definitely no (high risk of bias)
Prompting items and considerations	a. Adequate participation in the study by eligible persons (i.e., based on inclusion/exclusion criteria)	a. Adequate response rate for study participants (less than 5% considered small loss to follow-up and more than 20% considered significant)	a. A clear definition of the outcome is provided
	b. Description of the source population or population of interest	b. Unbiased and systematic attempts to collect information on participants who dropped out	b. Method of outcome measurement used is adequately valid and reliable
	c. Description of the baseline study sample of enrolled/included patients	c. Reasons for loss to follow-up are provided	c. The method and setting of outcome measurement is the same for all study participants
	d. Adequate description of the sampling frame and recruitment	d. Adequate description of participants lost to follow-up	d. The method and setting
	e. Adequate description of the period and place of recruitment	e. There are no important differences between participants who completed the study and those who did not	
	f. Adequate description of inclusion and exclusion criteria

**Table 2:** Definitions of levels of evidence for typical risk of broadly defined population used for risk of bias assessment [21, 22]

Quality level	Definition
High	We are very confident that the true prognosis (probability of future events) lies close to that of the estimate
Moderate	We are moderately confident that the true prognosis (probability of future events) is likely to be close to the estimate, but there is a possibility that it is substantially different
Low	Our confidence in the estimate is limited: the true prognosis (probability of future events) may be substantially different from the estimate
Very low	We have very little confidence in the estimate: the true prognosis (probability of future events) is likely to be substantially different from the estimate

### Data synthesis

Using the aggregated number of outcomes for each included study, summary estimates for proportions (i.e., all-cause mortality per major bleeds and fatal GI bleeding events per major GI bleeds) will be calculated using the random effects inverse variance method. A continuity correction of 0.5 will be used in studies with zero cell frequencies. A double arcsine transformation will be conducted prior to pooling proportions to stabilize estimates as there may be a range of proportions. As we expect the event rate to be over 1% (rates of 10% have been reported in some studies [14]) we believe this method to be statistically appropriate. The Wilson score method will be used to estimate 95% confidence intervals. Statistical heterogeneity will be evaluated using the *I*^2^ statistic and by providing 95% prediction intervals. We will assess the degree of heterogeneity to determine the degree to which pooling appears appropriate, for subgroups where *I*^2^ > 75% representing considerable heterogeneity we will not report pooled estimates. The potential for non-reporting bias and small study effect will be assessed for the primary study outcome by funnel plots representing the proportion of patients that died following a major GI bleed on a logit scale versus the inverse of sample size for each study [23], when 10 or more studies will be included in the meta-analysis. When asymmetry will be suggested by visual inspection, we will investigate asymmetry by Peter’s test using a weighted regression model with the logit of event rate as outcome variable, the inverse of sample size as predictor variable and the number of events and non-events to compute weights [24]. Analysis will be performed in R using the *meta* package for meta-analysis [25].

### Analysis of subgroups or subsets

Exploratory subgroup analyses will be conducted if feasible based on the following criteria: study design (prospective vs. retrospective), type of DOAC (apixaban, rivaroxaban, edoxaban, dabigatran), mechanism of DOAC (factor Xa inhibitors vs thrombin inhibitors), sex (male vs female), indication for anticoagulation (atrial fibrillation vs venous thromboembolism), concomitant antiplatelet use, and funding (i.e., industry vs non-industry vs none/not reported). We will also perform a sensitivity analysis based on the risk of bias for included studies as well as the information reported on GI vs extracranial bleeding.

## Discussion

This meta-analysis will synthesize evidence from studies on the risk of all-cause mortality after GI bleeding in patients on DOACs. The quality of evidence will be evaluated using a risk of bias tool adapted for this purpose. We do acknowledge that there may be substantial heterogeneity within the available data, and this may limit the number of studies that can be included in an ultimate meta-analysis.

Clinical discussions around the risks and benefits of anticoagulation focus on both prevention of vascular and bleeding risks. Bleeding related risk remains understudied, and a true understanding of mortality associated with GI bleeding would be of utility in clinical decision making [26]. In addition to discussions of anticoagulation initiation, restarting anticoagulation after a GI bleed is an area of ongoing study with substantial practice variation [27]. We anticipate the resulting evidence will provide specific data regarding the consequences of the most common complication of DOAC treatment to inform shared decision-making in routine clinical practice and future research questions.

## Data Availability

Not applicable.

## Appendix 1 Search strategy

**Table:**

#	Searches
1	thrombosis/
2	thromboembolism/
3	venous thromboembolism/
4	exp venous thrombosis/
5	exp pulmonary embolism/
6	((vein* or venous* or vena*) adj2 (thrombos* or thrombus* or thrombopro* or thrombotic* or thrombolic* or thromboemboli* or thrombo-emboli* or thrombophlebitis or thrombo-phlebitis)).ti,ab,kf
7	((pulmonary or lung or lungs) adj2 (embol* or microembol* or micro-embol* or thromboembol* or thrombo-embol*)).ti,ab,kf
8	(VTE or VTEs).ti,ab,kf
9	((blood or pulmonary or lung) adj3 clot*).ti,ab,kf
10	(phlebothrombos* or phlebo-thrombos*).ti,ab,kf
11	atrial fibrillation/
12	atrial flutter/
13	((atrial or auricular or atrium) adj4 (fibrillat* or flutter*)).ti,ab,kf
14	or/1–13
15	exp Gastrointestinal Hemorrhage/
16	((stomach or gastr* or duoden* or peptic* or esophag* or oesophag* or varices or variceal or ulcer* or ileum or ileal* or ileo* or jejun* or intestin* or bowel* or abdomen* or abdomin* or colon* or colorect* or rectal* or rectum* or GI or extra-cranial* or extracranial*) adj4 (bleed* or re-bleed* or rebleed* or blood or hemorrhag* or haemorrhag*)).ti,ab,kf
17	exp Gastrointestinal Tract/ and Hemorrhage/
18	(h?ematochezia* or h?ematemes* or mel?ena*).ti,ab,kf
19	or/15–18
20	Anticoagulants/
21	antithrombins/
22	Dabigatran/
23	rivaroxaban/
24	Factor Xa Inhibitors/
25	(oral anticoagula* or oral anti-coagula* or antithrombin* or anti-thrombin* or DOAC* or NOAC*).ti,ab,kf
26	((thrombin* or factor xa or factor 10a) adj2 inhibit*).ti,ab,kf
27	(apixaban* or eliquis* or eliques*).ti,ab,kf
28	(dabigatran* or pradaxa* or pradax* or rendix* or prazaxa*).ti,ab,kf
29	(edoxaban* or savaysa* or lixiana* or endoxaban* or roteas*).ti,ab,kf
30	(rivaroxaban* or xarelto* or throsaben* or xanirva*).ti,ab,kf
31	(betrixaban* or bevyxxa* or dexxience*).ti,ab,kf
32	or/20–31
33	14 and 19 and 32

## Appendix 2 Information extracted from studies

**Table:**

**Study-specific information**
▪ Year of publication, ▪ Enrolment period, ▪ Duration of follow-up ▪ Study design (randomized trial, retrospective cohort, prospective cohort), ▪ Source of funding (i.e., industry vs non-industry vs none/not reported), ▪ Total study participants ▪ Primary and secondary outcomes
**Patient demographics**
▪ Average age (mean with standard deviation and/or median with range or interquartile range) ▪ Number and proportion of males/females (sex) and/or men/women/non-binary (gender)
**Patient comorbidities**
▪ Malignancy or active cancer treatment ▪ Average creatinine (mean with standard deviation) ▪ Number and proportion of patients with severe renal impairment (e.g., creatinine clearance and/or eGFR < 30) ▪ Number and proportion of patients with a prior history of major bleeding ▪ Number and proportion of patients with prior GI bleeding ▪ Number and proportion of patients with chronic anemia ▪ Number and proportion of patients with documented atherosclerotic vascular disease (coronary artery disease, peripheral artery disease, cerebrovascular disease) ▪ Number and proportion of patients with prior VTE ▪ Number and proportion of patients with prior ischemic stroke
**Co-medications**
▪ Number and proportion of patients using single or dual antiplatelet therapies (e.g., acetylsalicylic acid, clopidogrel, prasugrel, ticagrelor) ▪ Number and proportion of patients using non-steroidal anti-inflammatory drugs
**Anticoagulation details**
▪ Number and proportion of patients receiving different DOAC agents (apixaban, rivaroxaban, edoxaban, dabigatran) ▪ Indication for anticoagulation
